# Quality care metrics for nurses working in general practice, mapping the evidence: a scoping review protocol

**DOI:** 10.12688/hrbopenres.13483.1

**Published:** 2022-01-21

**Authors:** Orla Loftus Moran, Mary Casey, Laserina O'Connor, Walter Cullen

**Affiliations:** 1 School of Nursing Midwifery and Health Systems, University College Dublin, Dublin, Ireland; 2 General Practice, Castlebar Family Practice, Castlebar, Ireland; 3 School of Medicine, Health Sciences, University College Dublin, Dublin, Ireland

**Keywords:** Scoping Review, Practice Nurse, General Practice Nurse, General Practice, Family Practice, Quality Care Metrics, Quality Care Indicators, Care Process.

## Abstract

**Background:** Irish general practice nursing roles have developed and grown exponentially in response to changing policy, clinical and workforce demands over the past three decades. However, as nursing care in general practice advances at pace, comprehensive evaluation of the general practice nurse (GPN) role has not been undertaken. Therefore, processes which enable robust data collection to carefully assess the role and facilitate development of services are required. Nursing quality care metrics (QCM) are an established mechanism which measure nursing care process, evaluate quality, and impact of care, and inform service development. The use of nursing QCM has been adopted within seven distinct healthcare settings in Ireland but not general practice. This scoping review is the first stage of a project which aims to inform development of QCM within Irish GPN settings.

**Aim: **To explore and map the literature regarding the use, application, and impact of nursing quality care metrics within a general practice, primary care setting.

**Methods:** The following five-stage methodological framework for scoping reviews proposed by Arksey and O’Malley will be used: (1) identifying the research question, (2) identifying relevant studies, (3) study selection, (4) charting/mapping the data and (5) collating, summarizing, and reporting results. The review will be conducted and reported in accordance with the PRISMA Extension for Scoping Reviews (PRISMA-ScR).

**Conclusions: **The focus of this scoping review relates to QCM which specifically measure the work of general practice nurses. It is envisioned that synthesis of international literature will give a broad perspective about QCM, their use in general practice or primary care settings, and enrich understanding of their development. It is anticipated that findings will provide key information to policy makers and health professionals interested in planning, strengthening, and delivering  primary care in Ireland.

## Introduction

The World Health Organisation (WHO) Astana declaration affirmed the role of primary care as the mainstay of efficient, effective, equitable health systems internationally. This declaration advocated that primary care be strengthened and supported with enhanced infrastructure and workforce capacity to enable its important role in disease prevention and health promotion ( World Health Organisation, 2018). General practice is considered the cornerstone of primary healthcare delivery in most European countries ( Kringos *et al.*, 2013). In Ireland, healthcare policy, government and healthcare providers are advancing and adopting reorientation of service delivery into the community and primary care ( Houses of the Oireachtas, 2017). This change comes at a critical time for Irish general practice, which is positioned at the centre of healthcare policy implementation. Currently demographic pressures, the rise of non-communicable diseases, multimorbidity, workforce capacity issues and the coronavirus disease 2019 (COVID-19) pandemic are contributing to increased pressure on general practice ( Crosbie *et al.*, 2020; McGlacken-Byrne *et al.*, 2021).

Primary care nursing worldwide is evolving in tandem with health policy reform and general practice nursing has emerged as a new generalist model of nursing positioned within primary care which is integral to the future delivery of healthcare ( Desborough *et al.*, 2016; Freund *et al.*, 2015; Groenewegen *et al.*, 2015; Halcomb *et al.*, 2017; International Council of Nurses, 2018; Lukewich *et al.*, 2020; Maier & Aiken, 2016; World Health Organisation, 2021). General practice is a core component of primary care services, led by general practitioners (GPs) it is the place of first contact for most patients in need of health services, and offers accessibility, continuity, and comprehensive, coordinated care. According to O’Dowd *et al.* (2017) the distinct role of general practice within primary care is an important consideration as the terms ‘general practice’ and ‘primary care’ are often incorrectly used interchangeably. A more detailed definition of general practice or family medicine is further summarised as *“General practice/family medicine is an academic and scientific discipline, with its own educational content, research, evidence base and clinical activity, and a clinical specialty orientated to primary care”* ( Mola *et al.* (2011) pp. 8).

Reflecting international trends Irish government health policy, *Sláintecare*, has embraced the concept of care reorientation from hospital-based models to primary care settings ( Houses of the Oireachtas, 2017). Fundamental to *Sláintecare* policy is implementation of a universal, single-tier health system, and delivery of high quality, safe care to patients in their own communities. Implementation of this policy is evident in recent agreements between healthcare authorities and general practice providers, including the introduction of entitlements to free general practice care for those aged under six and over seventy years of age ( Connolly & Wren, 2019; Health Service Executive, 2015b). In 2019, an agreement for reform and service development within general practice between government and general practice representatives, included a community-based structured chronic disease management programme (CDM) which became operational in 2020 ( Health Service Executive, 2019b). This is a first step in implementing an integrated approach to chronic disease care, and GPNs are established as critical to its successful rollout ( Collins & Homeniuk 2021; Darker *et al.,* 2015; Irish College of General Practitioners, 2021).

Irish GPNs lead and deliver a broad range of the healthcare services, including primary infant and adult immunisation programmes, cervical screening, health promotion and education interventions, disease surveillance, wound care, and a complex range of generalist nursing services ( Bury *et al.*, 2021; McCarthy *et al.*, 2012). Furthermore, GPNs adapted to challenges in delivery of services resulting from the COVID-19 pandemic, and were integral to continuation of services, particularly the delivery of the COVID-19 vaccination programme in general practice ( Homeniuk & Collins, 2021; Health Service Executive, 2021; Lowry Lehnen, 2021). International research demonstrates that mobilisation of GPN workforce and advanced GPN roles can strengthen general practice, whilst providing benefits and safe care for patients ( Halcomb & Ashley, 2019; Laurant *et al.*, 2018; Norful *et al.*, 2017; Poghosyan *et al.*, 2018), additionally this research suggests that GPN roles may be underutilized.

Enabling the provision of high quality healthcare is a core concern for healthcare authorities worldwide ( Health Information and Quality Authority (HIQA), 2012; World Health Organisation (WHO), 2006). In Ireland, *Sláintecare* policy endorses this drive towards accountability and performance within the health service. However, there is currently little evidence available on the quality of care, impact, and contribution of Irish GPNs to general practice. Nursing Quality Care Metrics (QCM) is a mechanism which can simultaneously address the issue of the provision of quality nursing care and GPN role evaluation. By compiling quantifiable measurements applicable to nursing care in general practice, development and improvements in nursing services and patient outcomes can be facilitated ( Health Service Executive, 2015a). This data will also give more detailed information on the nursing activities of the general practice nurse.

### Quality Care Metrics (QCM)

Quality Care Metrics is a method of measuring quality of care, placing safety and quality at the heart of service provision ( Foulkes, 2011). Maben *et al.* (2013) report on the increasing use of initiatives which measure quality care in nursing both internationally and within the UK. They counsel that the development of nursing metrics should align with ‘what matters most’ to patients. Furthermore, they observed practitioner’s acceptance and participation in QCM projects was enhanced through fostering understanding of the purpose and benefits of the process for nursing services, and patient care. The Office of the Nursing and Midwifery Services Director (ONMSD) has led QCM implementation and evaluation in Irish healthcare settings and define nursing QCMs as *“A measure of the quality of nursing and midwifery clinical care processes aligned to evidenced based standards and agreed through national consensus in healthcare settings in Ireland”* ( Health Service Executive (2015a) pp.10).

The Irish national QCM project adopted the Donabedian (1988) framework of quality care evaluation based on the structure, process, and outcome triad within which each element has a direct effect on the overall quality of care delivered. Within the Donabedian model, structure refers to the attributes specific to the healthcare working environment and characteristics of those who work within it such as education or experience, process examines how direct care is provided to patients from a technical and interpersonal perspective, and outcomes assess the end points of care achieved such as immunisation rates or patient experiences ( Hanae Ibn El *et al.*, 2013). The focus of QCM implemented by the ONMSD national project is on care specific to nursing processes, in other words scrutinising how nursing care is given, and how it is delivered. The areas which have adopted the use of metrics to date are acute care services, children’s services, intellectual disability services, older person services, mental health services, public health nursing services, and midwifery services ( Health Service Executive, 2019a).

In the UK, Griffiths *et al.* (2008) observed that development of QCM had been dominated by in-patient settings and this is reflected in the Irish experience. Notably, Griffiths *et al.* (2008) conclude that metric themes and indicators developed for acute care and inpatient settings can be modified and applied to other care settings. According to Haycock-Stuart & Kean (2012) community settings are a care environment within which nursing plays a significant but often invisible role and they acknowledge the need for evidence of quality care delivery in this setting. In Ireland a report into the development process for a primary care setting, Public Health Nursing QCM published in 2018 demonstrates the feasibility of QCM implementation in a community setting ( Health Service Executive, 2018). Utilising a rigorous process consisting of an initial systematic literature review, a two-round Delphi survey on identified metrics, followed by a two-round Delphi survey on associated indicators, and culminating with a stakeholder consensus meeting, 14 quality care process metrics and 69 associated indicators were developed. Central to the success of this project was establishment of a collaborative, participatory approach between key stakeholders, researchers, clinicians, and educators. This same process was used across all other six settings.

## Method

As outlined by Pollock *et al.* (2021), scoping reviews map and synthesise research, address broader topics and wide-ranging study designs, report on the depth and extent of the literature, identify knowledge gaps, and is an appropriate methodology to clarify nursing concepts. Given the broad nature of the question posed by this study and taking into consideration the little evidence which exists regarding QCMs use in general practice nursing, a scoping review is deemed the most appropriate methodology. A key aspect of scoping review is the extensive range of literature from both published and grey sources which can be included in the review. This literature synthesis of general practice nursing and QCMs will help to identify what is already known about the topic, it will reveal research deficits and knowledge gaps, informing and enabling future research.

This scoping review will adhere to the five stage framework first described by Arksey & O'Malley (2005) which are:

- Stage 1: identifying the research question.
- Stage 2: identification of relevant studies.
- Stage 3: study selection.
- Stage 4: charting the data.
- Stage 5: collating, summarising, and reporting the result.

### Stage 1: Identifying the research question

Joanna Briggs Institute (JBI) argues that development of the main research question is one of the most important steps to consider and advises using the Population-Concept-Context (PCC) framework to guide this ( Peters *et al.*, 2020a). This review aims to establish a comprehensive understanding of how nursing QCMs have been developed and implemented. It seeks to map the evidence and identify knowledge gaps focusing primarily on nursing in general practice. As usual, objectives will be used to demonstrate the relationship to the principal research question. This review will address the following question.

**Research question**

What is known about the use of nursing (Population) quality care process metrics (Concept) within general practice nursing (Context) settings?

**Review objectives**

• To **map the extent of evidence** available regarding nursing QCM pertinent to general practice nursing, primary care settings within the literature.
• To **identify methods** used to develop general practice nursing quality care metrics and other nursing metrics as appropriate
• To **ascertain the characteristics** of general practice nursing QCM.
• To determine if the literature **demonstrates implementation** of nursing QCM in general practice, and if their use provides advantages for patient outcomes and or service delivery?

### Stage 2: Identifying the relevant studies

In accordance with Peters *et al.* (2020b), the PCC framework will be used to develop search terms and align the eligibility criteria with study selection, and the assistance of a research librarian will be enlisted during all stages of this review. This will ensure an appropriate search strategy and database selection is in place ( Pollock *et al.*, 2021).

*Eligibility of population or types of participants:* This review will consider studies which focus on registered nurses working in general practice and primary care contexts. When discussing general practice nursing in an international perspective it is important to note there is some lack of uniformity in nomenclature ( Annells, 2007; Keleher *et al.*, 2009). This lack of uniformity will be addressed by inclusion and recognition of the broad range of terms used internationally when referring to nurses working within general practice or alongside a general practitioner or within family practice. The scope of the review will be limited to use of metrics in general practice nursing and relevant primary care settings.

*Concept:* Nursing quality care metrics are measurements of the quality of nurse performance when providing care to patients. Aligned with care, process metrics are indicators which give a framework within which nursing care can be measured ( Foulkes, 2011). For the purposes of this review, the definitions specific to QCM of nursing process and associated quality process indicators are as follows, *“Quality Care Process Metric: is a quantifiable measure that captures the quality in terms of how (or to what extent) nursing care is being done in relation to an agreed standard. Quality indicators associated with metrics are the tools or flags which demonstrate implementation of the nursing process.”* ( Health Service Executive (2018) pp.15). Therefore, in the context of this proposed review, a QCM looks at how and what nursing care processes are being performed by GPNs and what associated indicators were used to measure this care.

*Context, General practice setting:* Remaining cognisant of the distinctions between primary care and general practice, but to ensure a comprehensive synthesis of the literature, this review will aim to identify a broad scope of peer reviewed literature both published and grey which addresses implementation of nursing QCM in general practice, or equivalent primary care settings. Selection will be limited to publications in the English language. Conference abstracts will be excluded due to their limited data. The inception of Irish general practice nursing roles began in 1989 with the introduction of a subsidy from government to General Practitioners to support their employment therefore this study will limit itself to literature published since 1989 ( Department of Health, 1989). Table 1 details inclusion and exclusion criteria.

A three-step process as recommended by JBI will be implemented to carry out a comprehensive search of electronic databases, bibliographic references and grey literature in conjunction with a research librarian ( Peters *et al.*, 2020a).

**Table 1.** Inclusion and exclusion criteria. GPN=general practice nurse.

Criterion	Inclusion Criteria	Exclusion Criteria	Rationale
**Participants**	Registered nurses working in general practice and /or equivalent primary Care settings	Non-registered nurses or midwives	The focus of the protocol is on nursing
**Types of articles**	Peer reviewed empirical research journal publications, editorials and commentary articles, grey literature, academic theses, dissertations, reports. Guidelines and policy documents from Government and recognised professional nursing and medical bodies.	Conference abstracts.	To ensure that an extensive search as is reasonable, of available literature has been carried out, in keeping with the ethos of scoping reviews.
**Location**	All geographical locations.	No restriction.	To examine information from a broad range of locations.
**Time Period**	Literature published since 1989.	Research published prior to 1989, the initiation of formalised GPN roles in Ireland.	To assess how developments in GPN care processes have evolved over time.
**Language**	English language.	Languages other than English.

- **Step one** - carry out a preliminary search of the PubMed to identify papers relevant to the research question. Key words and search terms will be developed from this initial search and adapted to inform the final search strategy.
- **Step two** – a second search across all electronic databases will be carried out using the final search term strategy adopted for each specific database.
- **Step three** - This will be followed by a secondary search of the bibliographic references cited in the included studies.

Utilising the advice and collaboration of a research librarian it is proposed this review will search the following databases. These databases have been selected in view of their extensive repertoire and relevancy to nursing.

• Cochrane Database of Systematic Reviews
• PubMed, Biomedical and life sciences database
• CINAHL, Nursing and Allied Health ( CINAHL Plus)
• InterNurse Journals
• OpenGrey

### Stage 3: Study selection

An initial search of article titles and abstracts will be carried out by two researchers and a librarian to determine if the search critera captures data pertinent to the review question. Following this, any modifications to the search strategy will be agreed. Once the search process is completed the identified citations will be imported into the reference management system Endnote X9, with duplicates removed. This process will be guided by Peters (2017) stepwise method of source selection using Endnote. As described in Pollock *et al.* (2021) an ‘elaboration document’ detailing all included and excluded documents will be drafted to chart this process. Any discordance regarding article selection between initial researchers will be moderated by a third researcher who will review and advise on the articles eligibility for inclusion.

As JBI ( Peters *et al.*, 2020a) advises, pilot testing will be carried out prior to source selection the framework suggested is as follows.

• Random sample of 25 titles/abstracts is selected.
• The entire team screens these using the eligibility criteria and definitions/elaboration document.
• Team meets to discuss discrepancies and make modifications to the eligibility criteria and definitions/elaboration document.
• Team only starts screening when 75% (or greater) agreement is achieved.

In keeping with JBI guidance the Preferred Reporting Items for Systematic Reviews and Meta-Analysis extension for scoping reviews checklist (PRISMA-ScR) will be used to report the process of study selection ( Tricco *et al.*, 2018).

### Stage 4: Charting the data

In order to ensure selected articles are in keeping with inclusion and excluson criteria charting of data will be undertaken using a data extraction document as recomended by JBI ( Peters *et al.*, 2020a). This document will be created using Microsoft Excel software using the JBI data extraction tool template. It will be piloted for applicability to this study by two researchers and amended as necessary.

The proposed extraction document criteria for this study will include but will not be limited to the following: Article title; Authors; Journal; Publication date; Methodology; Location of Study; Population; Context; Concept and Key Findings applicable to review question identified? Examples of key finding would include, methods used to develop nursing metrics, characteristics of identified general practice nursing metrics, Impact on patient outcomes or health service delivery.

### Stage 5: Collating, summarising, and reporting the results

Inconsistent approaches in reporting scoping review findings have been highlighted by Bradbury-Jones *et al.* (2021) who have addressed this issue by developing the PAGER framework for improving the quality of scoping review reporting. The acronym PAGER refers to Patterns, Advances, Gaps, Evidence for Practice and Research recommendations. Therefore, to ensure careful and complete reporting of findings this metodology will be applied to reporting of this review. Initially a ‘Patterning chart’ outlining key themes discovered during this review will be generated. Following on from this reflective questions posed for each domain will be utilised to amplify findings and strengthen the quality of reporting of this review. Extracted data will be described in a thematic narrative report also providing a numerical analysis of the extent and nature of the identified studies. Reporting of this scoping review will utilise the Preferred Reporting Items for Systematic Reviews and Meta-Analysis extension for scoping reviews (PRISMA-ScR) ( Tricco *et al.*, 2018), and a flow diagram will also be produced to chart the process.

### Study status

At the time of publication of this protocol the study is at Stage 2, piloting and refining of search terms have begun. Full database searches will begin during January 2022.

## Discussion

The importance of reflecting on the rationale and motivation of any scoping study and not only the methodological process is highlighted by Levac *et al.* (2010). The key concept leading to this review is that nurses have an important, albeit hidden role in general practice. If the role is to be optimised analysis to clearly ascertain quality, value and outcomes is required. The literature on nurse QCM primarly focuses on inpatient or secondary care settings as Griffiths *et al.* (2008) has indicated, this review will provide insights into the use of nursing QCM within general practice, a primary care setting. This evidence synthesis will inform the next part of a research study, which is identification, development and refinement of relevant general practice nurse QCM. Dissemination of findings will contribute to the literature, and will be of value to nursing authorities, general practitioners, policy makers and academics who seek to develop and strengthen primary care. Findings of this scoping review will be submitted for publication in a peer reviewed journal, made available electronically and presented at conferences.

## Data availability

No data are associated with this article.
