# Quality care process metrics for nurses working in general practice, mapping the evidence: a scoping review protocol

**DOI:** 10.12688/hrbopenres.13483.2

**Published:** 2022-04-14

**Authors:** Orla Loftus Moran, Mary Casey, Laserina O'Connor, Walter Cullen

**Affiliations:** 1 School of Nursing Midwifery and Health Systems, University College Dublin, Dublin, Ireland; 2 General Practice, Castlebar Family Practice, Castlebar, Ireland; 3 School of Medicine, Health Sciences, University College Dublin, Dublin, Ireland

**Keywords:** Scoping Review, Practice Nurse, General Practice Nurse, General Practice, Family Practice, Quality Care Process Metrics, Quality Care Indicators.

## Abstract

**Background:** Irish general practice nursing roles have developed and grown exponentially in response to changing policy, clinical and workforce demands, this is reflective of international primary healthcare nursing trends. However, as nursing care in general practice advances, comprehensive evaluation of the general practice nurse (GPN) role has not been undertaken. Therefore, processes which enable robust data collection to assess the role and facilitate development of services are required. Nursing quality care metrics are an established mechanism which evaluate quality of care. Nursing quality care process metrics (QCP-Ms) specifically refer to measurement of care delivered directly to patients by nurses, benchmarking these interventions adherence to best practice guidance. The use of nursing metrics has been adopted within seven distinct healthcare settings in Ireland but not general practice. This scoping review is the first stage of a project which aims to inform development and implementation of QCP-Ms by Irish GPNs.

**Aim: **To explore and map the literature regarding the development and implementation of QCP-Ms within general practice settings.

**Methods:** The following five-stage methodological framework for scoping reviews proposed by Arksey and O’Malley will be used: (1) identifying the research question, (2) identifying relevant studies, (3) study selection, (4) charting/mapping the data and (5) collating, summarizing, and reporting results. The review will be conducted and reported in accordance with the PRISMA Extension for Scoping Reviews (PRISMA-ScR).

**Conclusions: **The focus of this scoping review relates to QCP-Ms which specifically measure the work of general practice nurses. It is envisioned that synthesis of international literature will give a broad perspective about nursing QCP-Ms, their use in general practice or primary healthcare settings, enriching understanding regarding their development. It is anticipated that findings will provide key information to policy makers and health professionals interested in planning, strengthening, and delivering primary healthcare.

## Introduction

The World Health Organisation (WHO) Astana declaration affirmed the role of primary care as the mainstay of efficient, effective, equitable health systems internationally. This declaration advocated that primary care be strengthened and supported with enhanced infrastructure and workforce capacity to enable its important role in disease prevention and health promotion ( World Health Organisation, 2018). General practice is considered the cornerstone of primary healthcare delivery in most European countries ( Kringos *et al.*, 2013). In Ireland, healthcare policy, government and healthcare providers are advancing and adopting reorientation of service delivery into the community and primary care ( Houses of the Oireachtas, 2017). This change comes at a critical time for Irish general practice, which is positioned at the center of healthcare policy implementation. Currently demographic pressures, the rise of non-communicable diseases, multimorbidity, workforce capacity issues and the coronavirus disease 2019 (COVID-19) pandemic are contributing to increased pressure on general practice ( Crosbie *et al.*, 2020; McGlacken-Byrne *et al.*, 2021).

Primary care nursing worldwide is evolving in tandem with health policy reform, general practice nursing has emerged as a new generalist model of nursing positioned within primary care which is integral to the future delivery of healthcare. The World Health Organization (WHO) highlight the positive benefits of generalist nurse practitioners in family practice and recommends governments enable optimization of autonomous practice for nurses working in primary health care services ( World Health Organisation, 2021). The prominent, impactful and evolving role of nurses in primary care internationally was outlined by WHO in a recent ‘State of the world's nursing 2020’ report ( World Health Organisation, 2020). Furthermore, The Lancet (2019) in an editorial describes the inestimable value of nursing and the untapped potential which nursing offers to strengthen primary healthcare services. More recently Casey *et al.* (2022) suggest that general practice nurses provide a valuable resource to general practice particularly in chronic disease management and indeed they may be practicing at an advanced nursing level. Primary healthcare workforce skill mix in six countries, USA, Canada, Australia, England, Germany, and Netherlands was examined by ( Freund *et al.*, 2015) who reported nurses as the major non physician workforce in primary care teams. A 2018 Cochrane systematic review ( Laurant *et al.*, 2018) demonstrated quality-of-care interventions by nurses in general practice to be at least as good as care from general practitioners. This systematic review analyzed a broad scope of research from UK, Netherlands, USA, Canada, Sweden, Spain, and South Africa, demonstrating the international experience and impact of nurses working within primary care settings. Australian studies by ( Desborough *et al.*, 2016; Halcomb & Ashley, 2019) demonstrate the evolving role and impact of GPNs, their importance to health promotion and chronic disease management care. These studies highlight increased patient satisfaction derived from continuity of care offered by GPNs. They find the high-quality care delivered by GPNs is associated with patient enablement, better outcomes, and that the role is underutilized. It is notable from preliminary searches of the literature that researchers have been working towards the development of standards for general practice nursing in many countries, including the UK, Australia, and Canada. ( Barrett *et al.*, 2021; Halcomb *et al.*, 2017; Lukewich *et al.*, 2020). This demonstrates an imperative internationally to measure the delivery of GPN care, and articulate the unique generalist aspects of the role. The recent Sonnet report published on behalf of NHS England and NHS Improvement evaluates the value which GPNs deliver to primary healthcare in the UK ( Clifford *et al*., 2021), it describes nursing in general practice as a clear, identifiable discipline, which brings significant value to primary healthcare. This report stresses that without recognition, investment, training and establishment of a distinct career pathway for GPNs the role may flounder. In Canada Lukewich *et al*. (2021) echoed these concerns and draw attention to obstacles to progression which general practice nursing faces emphasizing the need for research and support of leadership development among GPNs.

General practice is a core component of primary care services, led by general practitioners (GPs) it is the place of first contact for most patients in need of health services, and offers accessibility, continuity, and comprehensive, coordinated care. According to O’Dowd *et al.* (2017) the distinct role of general practice within primary care is an important consideration as the terms ‘general practice’ and ‘primary care’ are often incorrectly used interchangeably. A more detailed definition of general practice or family medicine is further summarised as *“General practice/family medicine is an academic and scientific discipline, with its own educational content, research, evidence base and clinical activity, and a clinical specialty orientated to primary care”* ( Mola *et al.* (2011) pp. 8). The World Organisation of Family Doctors (WONCA) represents a global workforce of over 500,000 general practitioner, family doctors. In 2019 WONCA endorsed and authored the Albuquerque Statement which specifically seeks to advance and advocate for nurses working within rural primary healthcare settings ( Kenkre & Wynn-Jones, 2020). The Albuquerque statement recognises the importance of generalism in nursing, it commits to supporting research and data collection which will enhance understanding of the work of family practice and rural community nursing roles.

Reflecting international trends Irish government health policy, *Sláintecare*, has embraced the concept of care reorientation from hospital-based models to primary care settings ( Houses of the Oireachtas, 2017). Fundamental to *Sláintecare* policy is implementation of a universal, single-tier health system, and delivery of high quality, safe care to patients in their own communities. Implementation of this policy is evident in recent agreements between healthcare authorities and general practice providers, including the introduction of entitlements to free general practice care for those aged under six and over seventy years of age ( Connolly & Wren, 2019; Health Service Executive, 2015b). In 2019, an agreement for reform and service development within general practice between government and general practice representatives, included a community-based structured chronic disease management programme (CDM) which became operational in 2020 ( Health Service Executive, 2019b). This is a first step in implementing an integrated approach to chronic disease care, and GPNs are established as critical to its successful rollout ( Collins & Homeniuk, 2021; Darker *et al.,* 2015; Irish College of General Practitioners, 2021).

Irish GPNs lead and deliver a broad range of the healthcare services, including primary infant and adult immunisation programmes, cervical screening, health promotion and education interventions, disease surveillance, acute and minor illness triage, and management, wound care, and a complex range of generalist nursing services ( Bury *et al.*, 2021; Casey *et al*., 2022; McCarthy *et al.*, 2012). Furthermore, GPNs adapted to challenges in delivery of services resulting from the COVID-19 pandemic, and were integral to continuation of services, particularly the delivery of the COVID-19 vaccination programme in general practice ( Homeniuk & Collins, 2021; Health Service Executive, 2021; Lowry Lehnen, 2021). International research demonstrates that mobilisation of GPN workforce and advanced GPN roles can strengthen general practice, whilst providing benefits and safe care for patients ( Halcomb & Ashley, 2019; Laurant *et al.*, 2018; Norful *et al.*, 2017; Poghosyan *et al.*, 2018), additionally this research suggests that GPN roles may be underutilized.

Enabling the provision of high-quality healthcare is a core concern for healthcare authorities worldwide ( Health Information and Quality Authority (HIQA), 2012; World Health Organisation (WHO), 2006). In Ireland, *Sláintecare* policy endorses this drive towards accountability and performance within the health service. However, there is currently little evidence available on the quality of care and contribution of Irish GPNs to general practice. Nursing quality care process metrics is a mechanism which can simultaneously address the issue of the provision of quality nursing care and GPN role evaluation. By compiling quantifiable measurements applicable to nursing care in general practice, development and improvements in nursing services and patient outcomes can be facilitated ( Health Service Executive, 2015a). This data will also give more detailed information on the nursing activities of the general practice nurse.

### Nursing Quality Care Process Metrics

Quality care metrics is a method of measuring quality of care, placing safety and quality at the heart of service provision, nursing metrics were first introduced the USA in the early 1990s and were subsequently developed for use in the UK in 2008 ( Foulkes, 2011). Maben *et al.* (2013) report on the increasing use of initiatives which measure quality care in nursing both internationally and within the UK. They counsel that the development of nursing metrics should align with ‘what matters most’ to patients. Furthermore, they observed practitioner’s acceptance and participation in nursing metrics projects were enhanced through fostering understanding of the purpose and benefits of the process for nursing services, and patient care. The Office of the Nursing and Midwifery Services Director (ONMSD) has led QCP-Ms implementation and evaluation in Irish healthcare settings and define nursing QCP-Ms as *“A measure of the quality of nursing and midwifery clinical care processes aligned to evidenced based standards and agreed through national consensus in healthcare settings in Ireland”* ( Health Service Executive (2015a) pp.10).

The Irish national QCP-Ms project adopted the Donabedian (1988) framework of quality care evaluation based on the structure, process, and outcome triad within which each element has a direct effect on the overall quality of care delivered. Within the Donabedian model, structure refers to the attributes specific to the healthcare working environment and characteristics of those who work within it such as education or experience, process examines how direct care is provided to patients from a technical and interpersonal perspective, and outcomes assess the end points of care achieved such as immunisation rates or patient experiences ( Hanae Ibn El *et al.*, 2013). The focus of metrics implemented by the ONMSD national project is on care specific to nursing processes, in other words scrutinizing how nursing care is given, and how it is delivered. The areas which have adopted the use of metrics to date are acute care services, children’s services, intellectual disability services, older person services, mental health services, public health nursing services, and midwifery services ( Health Service Executive, 2019a).

In the UK, Griffiths *et al.* (2008) observed that development of nursing metrics had been dominated by in-patient settings and this is reflected in the Irish experience. Notably, Griffiths *et al.* (2008) conclude that metric themes and indicators developed for acute care and inpatient settings can be modified and applied to other care settings. According to Haycock-Stuart & Kean (2012) community settings are a care environment within which nursing plays a significant but often invisible role and they acknowledge the need for evidence of quality care delivery in this setting. In Ireland a report into the development process for a primary care setting, Public Health Nursing QCP-Ms published in 2018 demonstrates the feasibility of their implementation in a community setting ( Health Service Executive, 2018). Utilising a rigorous process consisting of an initial systematic literature review, a two-round Delphi survey on identified metrics, followed by a two-round Delphi survey on associated indicators, and culminating with a stakeholder consensus meeting, 14 quality care process metrics and 69 associated indicators were developed. Some metrics identified pertinent to public health nursing, which may be applicable to general practice nursing included wound assessment and health promotion. Central to the success of this project was establishment of a collaborative, participatory approach between key stakeholders, researchers, clinicians, and educators. This same process was used to determine the QCP-Ms for all other six settings (( O'Connor *et al.*, 2021)

Development of nurse sensitive metrics specifically pertinent to ambulatory care, an equivalent of general practice, is being undertaken in the USA ( Alley *et al.*, 2021; Mastal *et al.*, 2016; Start *et al.*, 2018) *The Primary Care and Ambulatory Specialty Institute* (PCASI) established in 2016 within a New York area health system, spearheaded an initiative to identify nurse sensitive metrics and develop a data dashboard application to enable recording of ambulatory nurse care interventions. Examples of measures or metrics identified and integrated on the PCASI dashboard include Incidence of (hospital) readmissions, High blood pressure and follow up care screening, Pain assessment and follow up, BMI screening Adult and Paediatric, Controlled hypertension, Uncontrolled Hemoglobin A1c. A telehealth disposition metric was added in response to the implementation of remote nursing care via telephone during the COVID 19 pandemic. The range of metrics identified demonstrate the generalist nature of GPN, ambulatory care nurses. 

## Method

As outlined by Pollock *et al.* (2021), scoping reviews map and synthesize research, address broader topics and wide-ranging study designs, report on the depth and extent of the literature, identify knowledge gaps, and is an appropriate methodology to clarify nursing concepts. Given the broad nature of the question posed by this study and taking into consideration the little evidence which exists regarding QCP-Ms use in general practice nursing, a scoping review is deemed the most appropriate methodology. A key aspect of scoping review is the extensive range of literature from both published and grey sources which can be included in the review. This literature synthesis of general practice nursing and QCP-Ms will help to identify what is already known about the topic, it will reveal research deficits and knowledge gaps, informing and enabling future research.

This scoping review will adhere to the five stage framework first described by Arksey & O'Malley (2005) which are:

- Stage 1: identifying the research question.
- Stage 2: identification of relevant studies.
- Stage 3: study selection.
- Stage 4: charting the data.
- Stage 5: collating, summarising, and reporting the result.

### Stage 1: Identifying the research question

Joanna Briggs Institute (JBI) argues that development of the main research question is one of the most important steps to consider and advises using the Population-Concept-Context (PCC) framework to guide this ( Peters *et al.*, 2020a). This review aims to establish a comprehensive understanding of how nursing QCP-Ms have been developed and implemented. It seeks to map the evidence and identify knowledge gaps focusing primarily on nursing in general practice. As usual, objectives will be used to demonstrate the relationship to the principal research question. This review will address the following question.

**Research question**

What is known about the use of nursing (Population) quality care process metrics (Concept) within general practice nursing (Context) settings?

**Review objectives**

- To **map the extent of evidence** available regarding nursing QCP-Ms pertinent to general practice nursing, primary care settings within the literature.
- To **identify methods** used to develop general practice nursing quality care metrics and other nursing metrics as appropriate
- To **ascertain the characteristics** of general practice nursing QCP-Ms.
- To determine if the literature **demonstrates implementation** of nursing QCP-Ms in general practice?

### Stage 2: Identifying the relevant studies

In accordance with Peters *et al.* (2020b), the PCC framework will be used to develop search terms and align the eligibility criteria with study selection, and the assistance of a research librarian will be enlisted during all stages of this review. This will ensure an appropriate search strategy and database selection is in place ( Pollock *et al.*, 2021).

*Eligibility of population or types of participants:* This review will consider studies which focus on registered nurses working in general practice and primary care contexts. When discussing general practice nursing in an international perspective it is important to note there is some lack of uniformity in nomenclature ( Annells, 2007; Keleher *et al.*, 2009). Barrett *et al.* (2021) caried out a comprehensive analysis of job titles and education requirements specific to registered nurses in primary care internationally, this analysis demonstrated the diversity of unofficial terms for GPNs. This lack of uniformity will be addressed by inclusion and recognition of the broad range of terms used internationally when referring to nurses working within general practice, alongside a general practitioner or within family practice and ambulatory care. As established by Barrett *et al.* (2021) “registered nurse” is the most common protected title internationally, this scoping review will consider only GPNs who hold a ‘protected’ nursing title in their relevant country. The scope of this review will be limited to use of metrics in general practice nursing and relevant primary care settings, nurses practicing at all levels including advanced nurse practitioners will be considered.

*Concept:* Nursing quality care metrics are measurements of the quality of nurse performance when providing care to patients. Aligned with care, process metrics are indicators which give a framework within which nursing care can be measured ( Foulkes, 2011). For the purposes of this review, the definitions specific to QCP-Ms and associated process metrics are as follows, *“Quality Care Process Metric: is a quantifiable measure that captures the quality in terms of how (or to what extent) nursing care is being done in relation to an agreed standard. Quality indicators associated with metrics are the tools or flags which demonstrate implementation of the nursing process.”* ( Health Service Executive (2018) pp.15). Therefore, in the context of this proposed review, a QCP-Ms looks at how and what nursing care processes are being performed by GPNs and what associated indicators were used to measure this care.

*Context, General practice setting:* Remaining cognisant of the distinctions between primary care and general practice, but to ensure a comprehensive synthesis of the literature, this review will aim to identify a broad scope of peer reviewed literature both published and grey which addresses implementation of nursing QCP-Ms in general practice, or equivalent primary care settings. Selection will be limited to publications in the English language. Conference abstracts will be excluded due to their limited data. The inception of Irish general practice nursing roles began in 1989 with the introduction of a subsidy from government to General Practitioners to support their employment therefore this study will limit itself to literature published since 1989 ( Department of Health, 1989). Table 1 details inclusion and exclusion criteria.

**Table 1.** Inclusion and exclusion criteria. GPN=general practice nurse.

Criterion	Inclusion Criteria	Exclusion Criteria	Rationale
**Participants**	Registered nurses working in general practice and/or equivalent primary healthcare settings	Non-registered nurses or midwives	The focus of the protocol is on nursing care
**Types of** ** articles**	Peer reviewed empirical research; including Qualitative, Quantitative, Mixed methods and Action Research studies. Grey literature, academic theses, dissertations, reports. Guidelines and policy documents from Government and recognised professional nursing and medical bodies.	Conference abstracts. Editorials and commentary articles,	To ensure that an extensive search as is reasonable, of available literature has been carried out, in keeping with the ethos of scoping reviews.
**Location**	All geographical locations.	No restriction.	To examine information from a broad range of locations.
**Time Period**	Literature published since 1989.	Research published prior to 1989, the initiation of formalised GPN roles in Ireland.	To assess how developments in GPN care processes have evolved over time. This timescale also reflects the first reports of adaptation and introduction of metrics specific to the measurement of nursing quality care.
**Language**	English language.	Languages other than English.	

A three-step process as recommended by JBI will be implemented to carry out a comprehensive search of electronic databases, bibliographic references, key author, relevant journals and grey literature in conjunction with a research librarian ( Peters *et al.*, 2020a).

- **Step one** - carry out a preliminary search of the PubMed to identify papers relevant to the research question. Key words and search terms will be developed from this initial search and adapted to inform the final search strategy.
- **Step two** – a second search across all electronic databases will be carried out using the final search term strategy adopted for each specific database.
- **Step three** - This will be followed by a secondary search of the bibliographic references cited in the included studies.

Utilising the advice and collaboration of a research librarian it is proposed this review will search the following databases. These databases have been selected in view of their extensive repertoire and relevancy to nursing.

- Embase
- PubMed, Biomedical and life sciences database
- CINAHL, Nursing and Allied Health ( CINAHL Plus)
- Web of Science

### Stage 3: Study selection

An initial search of article titles and abstracts will be carried out by two researchers and a librarian to determine if the search criteria capture data pertinent to the review question. Following this, any modifications to the search strategy will be agreed. Once the search process is completed the identified citations will be imported into the reference management system Endnote X9, with duplicates removed. Covidence software tool will be utilized to carry out screening of citations, abstracts, full text studies and data extraction. Any discordance regarding article selection between initial researchers will be moderated by a third researcher who will review and advise on the article’s eligibility for inclusion.

As JBI ( Peters *et al.*, 2020a) advises, pilot testing will be carried out prior to source selection the framework suggested is as follows.

- Random sample of 25 titles/abstracts is selected.
- The entire team screens these using the eligibility criteria and definitions/elaboration document.
- Team meets to discuss discrepancies and make modifications to the eligibility criteria and definitions/elaboration document.
- Team only starts screening when 75% (or greater) agreement is achieved.

In keeping with JBI guidance the Preferred Reporting Items for Systematic Reviews and Meta-Analysis extension for scoping reviews checklist (PRISMA-ScR) will be used to report the process of study selection ( Tricco *et al.*, 2018).

### Stage 4: Charting the data

In order to ensure selected articles are in keeping with inclusion and exclusion criteria charting of data will be undertaken using a data extraction document as recommended by JBI ( Peters *et al.*, 2020a). This document will be created using Microsoft Excel software using the JBI data extraction tool template. It will be piloted for applicability to this study by two researchers and amended as necessary.

The proposed extraction document criteria for this study will include but will not be limited to the following: Article title; Authors; Journal; Publication date; Methodology; Location of Study; Population; Context; Concept and Key Findings applicable to review question identified? Examples of key finding would include, methods used to develop nursing process metrics, implementation and characteristics of identified general practice nursing metrics.

### Stage 5: Collating, summarising, and reporting the results

Inconsistent approaches in reporting scoping review findings have been highlighted by Bradbury-Jones *et al.* (2021) who have addressed this issue by developing the PAGER framework for improving the quality of scoping review reporting. The acronym PAGER refers to Patterns, Advances, Gaps, Evidence for Practice and Research recommendations. Therefore, to ensure careful and complete reporting of findings this methodology will be applied to reporting of this review. Initially a ‘Patterning chart’ outlining key themes discovered during this review will be generated. Following on from this, reflective questions posed for each domain will be utilised to amplify findings and strengthen the quality of reporting of this review. Extracted data will be described in a thematic narrative report also providing a numerical analysis of the extent and nature of the identified studies. Reporting of this scoping review will utilise the Preferred Reporting Items for Systematic Reviews and Meta-Analysis extension for scoping reviews (PRISMA-ScR) ( Tricco *et al.*, 2018), and a flow diagram will also be produced to chart the process.

### Study status

At the time of publication of this protocol the study is at Stage 2, piloting and refining of search terms have begun.

## Discussion

The importance of reflecting on the rationale and motivation of any scoping study and not only the methodological process is highlighted by Levac *et al.* (2010). The key concept leading to this review is that nurses have an important, albeit hidden role in general practice. If the role is to be optimised analysis to clearly ascertain quality, value and outcomes is required. The literature on nurse metrics primarily focuses on inpatient or secondary care settings as Griffiths *et al.* (2008) has indicated, this review will provide insights into the use of nursing QCP-Ms within general practice, a primary care setting. This evidence synthesis will inform the next part of a research study, which is identification, development, and refinement of relevant general practice nurse QCP-Ms. A limitation of this scoping review is that it does not include quality assessment of included studies but utilising the PAGER framework for reporting will ensure a consistent clear approach to analysis and reporting of the review. A strength of the review is its unique nature and potential value in enabling meaningful data to articulate the contribution of GPNs to quality patient care. Dissemination of findings will contribute to the literature, and will be of value to nursing authorities, general practitioners, policy makers and academics who seek to develop and strengthen primary care. Findings of this scoping review will be submitted for publication in a peer reviewed journal, made available electronically and presented at conferences.

## Data availability

No data are associated with this article.
