# Supplementary material for: Quality care process metrics for nurses working in general practice, mapping the evidence: a scoping review protocol
Source: HRB Open Res. 2022 Apr 14;5:10. Originally published 2022 Jan 21. [Version 2] doi: 10.12688/hrbopenres.13483.2 (PMC9024129; doi:10.12688/hrbopenres.13483.2)
Supplement: Peer review history

# Peer review: Reviewer response for version 2

**DOI:** 10.21956/hrbopenres.14794.r31877

**Published:** 2022-04-21

**Authors:** Julia Lukewich

**Affiliations:** 1 Faculty of Nursing, Memorial University, Newfoundland, Canada

Revisions are appropriate.

Is the study design appropriate for the research question?

Partly

Is the rationale for, and objectives of, the study clearly described?

Yes

Are sufficient details of the methods provided to allow replication by others?

Partly

Are the datasets clearly presented in a useable and accessible format?

Not applicable

Reviewer Expertise:

Primary care/family practice nursing

I confirm that I have read this submission and believe that I have an appropriate level of expertise to confirm that it is of an acceptable scientific standard.

# Peer review: Reviewer response for version 2

**DOI:** 10.21956/hrbopenres.14794.r31876

**Published:** 2022-04-20

**Authors:** Anne Parkinson

**Affiliations:** 1 Department of Health Services Research and Policy, Australian National University, Acton, ACT, Australia

The authors have adequately addressed my comments and justified their reasoning. As always, resources determine what can and cannot be done with regard to research and choices must always be made. I believe the paper is much improved after peer review and am happy to recommend the paper be indexed in its current form.

Is the study design appropriate for the research question?

Yes

Is the rationale for, and objectives of, the study clearly described?

Yes

Are sufficient details of the methods provided to allow replication by others?

Yes

Are the datasets clearly presented in a useable and accessible format?

Not applicable

Reviewer Expertise:

Health services research, primary care, patient and public involvement, co-production, health experience

I confirm that I have read this submission and believe that I have an appropriate level of expertise to confirm that it is of an acceptable scientific standard.

# Peer review: Reviewer response for version 1

**DOI:** 10.21956/hrbopenres.14701.r31645

**Published:** 2022-03-21

**Authors:** Anne Parkinson

**Affiliations:** 1 Department of Health Services Research and Policy, Australian National University, Acton, ACT, Australia

This work aims to inform the development of quality care metrics (QCM) to measure the work of general practice nurses in Ireland. This is a novel application of QCM in Ireland. It has been adopted for use in acute care services, children’s services, intellectual disability services, older person services, mental health services, public health nursing services, and midwifery services, but not for general practice.

 This is a topic of value as primary care is the foundation of a successful healthcare system and general practice plays an important role in providing care and support to a diverse range of patients and their families and carers. The expanding scope of general practice nurses in line with increasing workforce demands requires monitoring and assessment to ensure the quality of care.

- A scoping review is an appropriate first step to inform how to target future research on this topic and determine if it is needed. The introduction reads well, however, it could benefit from expansion to briefly consider the international situation (role of GPNs) as you will be drawing on their experiences in your literature search.
- In the Methods, I suggest you use the version of Arksey and O'Malley enhanced by Levac *et al.* ^1^ that adds a sixth step, consulting relevant stakeholders (e.g. GPNs), which adds to rigour. I find this can be valuable as it incorporates the expertise of stakeholders and aids validation of the findings. If you do this, you will need to identify stakeholders you plan to consult and how you will collect, analyse and integrate the data in your work.
- There is no mention in the methods about the type of studies to be included (e.g qualitative, quantitative, mixed methods) and how you will determine whether to include a mixed-methods study. This needs further explanation in the manuscript.
- Also, how will you assess the quality of included studies? For example, CASP for qualitative studies, MMAT for mixed-methods. There are many options. This needs further explanation in the manuscript.
- I think it would be useful to reflect on the strengths and limitations of the study. For an example of a relevant protocol that incorporates this, see Jolley *et al*. ^2^.
- Stage 5: typo “methodology”.
- As per Reviewer 1, you might like to consider using Covidence software if you have access to it for organising your results and screening as it enables multiple reviewers to work together https://www.covidence.org/.
- Study status: I find it a little odd that the study has begun as it is a protocol. I respect your honesty but it is confusing because you now have search terms finalised, etc.

Is the study design appropriate for the research question?

Yes

Is the rationale for, and objectives of, the study clearly described?

Yes

Are sufficient details of the methods provided to allow replication by others?

Yes

Are the datasets clearly presented in a useable and accessible format?

Not applicable

Reviewer Expertise:

Health services research, primary care, patient and public involvement, co-production, health experience

I confirm that I have read this submission and believe that I have an appropriate level of expertise to confirm that it is of an acceptable scientific standard, however I have significant reservations, as outlined above.

## References

[1] : Scoping studies: advancing the methodology. *Implement Sci* .2010;5: 10.1186/1748-5908-5-69 69 10.1186/1748-5908-5-69 20854677PMC2954944

[2] : Protocol for a scoping review study to identify and classify patient-centred quality indicators. *BMJ Open* .2017;7(1) : 10.1136/bmjopen-2016-013632 10.1136/bmjopen-2016-013632 PMC522371428057655

# Response

**Published:** 2022-04-08

**Authors:** Orla Loftus Moran

**Affiliations:** Castlebar Family Practice, Ireland

We are very grateful for your time and expertise in reviewing this scoping review protocol. Amendments have been made in view of your insight and suggestions. We have replied to your comments, numbered below. 

**1. The introduction reads well, however, it could benefit from expansion to briefly consider the international situation (role of GPNs) as you will be drawing on their experiences in your literature search.**

 Thank you for highlighting this, discussion and references regarding international studies have been included in the Introduction section of the review. We agree that this gives a broader understanding of the importance of the role and its relevance to changing healthcare policy focus worldwide. As outlined in a response to a previous review, international research draws attention to the positive impact and potential of the role, but also identifies barriers to progress and the need for research to inform the future of nursing in general practice.

**2. In the Methods, I suggest you use the version of Arksey and O'Malley enhanced by Levac et al.1 that adds a sixth step, consulting relevant stakeholders (e.g. GPNs), which adds to rigour. I find this can be valuable as it incorporates the expertise of stakeholders and aids validation of the findings. If you do this, you will need to identify stakeholders you plan to consult and how you will collect, analyse and integrate the data in your work.**

 This is a very important aspect of the review that the research team considered at length. As the primary reviewer is self-funded and limited by resources and time due to clinical work commitments, it was agreed to continue with the 5 stage model for the protocol. This may be open to reevaluation when advancing to the full scoping review.

**3. There is no mention in the methods about the type of studies to be included (e.g qualitative, quantitative, mixed methods) and how you will determine whether to include a mixed-methods study.**

 We propose to include the following types of study designs. Peer-reviewed empirical research, including qualitative, quantitative, mixed methods, and action research studies. This is appropriate to the broad nature of scoping reviews within which study quality or methodological limitations are usually not appraised ^1^. This is outlined in Table 1. Inclusion and exclusion criteria.

**4. How will you assess the quality of included studies?**

 It is our understanding that scoping reviews do not seek to report on the quality of evidence as reported by Munn ^1^ and referred to in point 3 above. However, the PAGER framework for improving the quality of scoping review reporting will be used to ensure a consistent clear approach to analysis and reporting of the review findings ^2^.

**5. It is useful to reflect on the strengths and limitations of the study.**

 We have included reflections on the strengths and limitations of this protocol in the discussion section of the document.

**6. Stage 5: typo “methodology”.**

 Thank you – this is corrected.

**7. Consider using Covidence software?**

 We have now proposed the use of Covidence, this is referenced in the ‘ Stage 3: Study selection’ section of the document.

**8. I find it a little odd that the study has begun as it is a protocol. I respect your honesty, but it is confusing because you now have search terms finalised, etc**

 Apologies for any confusion caused regarding the stage of the study. The review is currently in a preliminary phase; to date refinement of the scoping document based on reviewer commentary is being undertaken. Liaison is taking place with a librarian and piloting of search terms is being carried out to ensure an appropriate search strategy is in place for the full review.

**References.**

 1.  Munn, Z., et al., *Systematic review or scoping review? Guidance for authors when choosing between a systematic or scoping review approach.* BMC Medical Research Methodology, 2018. **18**(1): p. 143.

 2.  Bradbury-Jones, C., et al., *Scoping reviews: the PAGER framework for improving the quality of reporting.* International Journal of Social Research Methodology, 2021: p. 1-14.

# Peer review: Reviewer response for version 1

**DOI:** 10.21956/hrbopenres.14701.r31343

**Published:** 2022-02-22

**Authors:** Julia Lukewich

**Affiliations:** 1 Faculty of Nursing, Memorial University, Newfoundland, Canada

This is a novel and important scoping review which aims to inform development of quality care metrics (QCM) within Irish general practice settings.

- This is a topic that will be attractive to an international reader. Within your Introduction, please broaden the discussion beyond Irish general practice/GPNs, e.g. what about this role in other countries such as Australia, Canada, New Zealand?
- Within abstract, if there is space within journal guidelines/word restrictions, additional details regarding the methods should be provided, e.g. anticipated date of search. 
- Is there a difference between ‘quality care process metrics’ and ‘QCM’? These are both used interchangeably in the manuscript. I recommend being consistent.
- With respect to your search strategy, did you consider search PsycINFO, Embase, Joanna Briggs Institute (JBI) Library of Systematic Reviews, and/or ProQuest Dissertations and Theses? It is possible that relevant nursing literature might be located within these databases. Will you conduct key author/journal searches?
- Under ‘Eligibility of population or types of participants’, Barret *et al*.'s (2021 ^1^)  article may be useful when discussing lack of uniformity in nomenclature. 
- What types of study designs are considered for inclusion? You mention broad scope of peer reviewed literature and that conference abstracts will be excluded. However, I do not see anything specifically referring to study type/design.
- Are you able to provide examples of quality care metrics?
- Please clarify if advanced practice nurses/nurse practitioners or other classes of registered nursing are included/excluded within your review.
- One of your objectives is to examine the use of QCM in providing advantages for patient outcomes and/or service delivery – however, your inclusion/exclusion criteria do not include anything related to reporting of outcomes within studies. Also, wouldn't this objective lend itself to a systematic review rather then a scoping review?
- Have you considered using Covidence (or similar software) to manage references/screening process?
- Please comment on how the quality of studies will be assessed.

Is the study design appropriate for the research question?

Partly

Is the rationale for, and objectives of, the study clearly described?

Yes

Are sufficient details of the methods provided to allow replication by others?

Partly

Are the datasets clearly presented in a useable and accessible format?

Not applicable

Reviewer Expertise:

Primary care/family practice nursing

I confirm that I have read this submission and believe that I have an appropriate level of expertise to confirm that it is of an acceptable scientific standard, however I have significant reservations, as outlined above.

## References

[1] : Job titles and education requirements of registered nurses in primary care: An international document analysis. *International Journal of Nursing Studies Advances* .2021;3: 10.1016/j.ijnsa.2021.100044 10.1016/j.ijnsa.2021.100044 PMC1108040038746715

# Response

**Published:** 2022-04-08

**Authors:** Orla Loftus Moran

**Affiliations:** Castlebar Family Practice, Ireland

We are very grateful for your time and expertise in reviewing our scoping review protocol, your critique is constructive and valuable. The scoping review has been revised in response to your review. We have replied to your comments, numbered below.

**1. Within your Introduction, please broaden the discussion beyond Irish general practice/GPNs, e.g. what about this role in other countries such as Australia, Canada, New Zealand?**

 Your suggestion to broaden the discussion to include an international context has strengthened the arguments in favour of carrying out this review. The international dimension has been addressed in the Introduction section with the addition of relevant articles which outline important findings regarding general practice nursing. This research draws attention to the positive impact and potential of the role, but also identifies barriers to progress. These studies highlight many common themes of an impactful but invisible, undervalued workforce, requiring leadership and robust research which will inform policymakers and healthcare strategists.

**2. Within abstract, if there is space within journal guidelines/word restrictions, additional details regarding the methods should be provided, e.g. anticipated date of search**

 Unfortunately, the constraints of the word count preclude this.

**3. Is there a difference between ‘quality care process metrics’ and ‘QCM’? These are both used interchangeably in the manuscript** **.**

 Thank you for noting this. Quality care metrics is a broad term that may include three types of healthcare quality assessment: structure, process, and outcomes. This is discussed in the ‘Nursing Quality Care Process Metrics’ section of the review. Quality care process metrics (QCP-Ms) measure specifically the quality of care delivered by nurses. This concept has now been clarified, addressed, and integrated into the title and throughout the document. A working definition of quality care process metrics (QCP-Ms) is included within the ‘Stage 2: Identifying the relevant studies, Concept’ section of the document.

**4. With respect to your search strategy, did you consider search PsycINFO, Embase, Joanna Briggs Institute (JBI) Library of Systematic Reviews, and/or ProQuest Dissertations and Theses?**

 As a self-funded researcher, limitations of resources & time would not allow searching all these databases; however, we have included Embase and Web of Science. Cochrane database is indexed on PubMed and Web of Science, therefore any relevant studies will be identified within these databases and included in the review.

**5. Will you conduct key author/journal searches?**

 Yes, reference to this is included in section ‘Stage 2: Identifying the relevant studies’.

**6. Under ‘Eligibility of population or types of participants’, Barret et al.'s (2021)  article may be useful when discussing lack of uniformity in nomenclature.**

 Thank you for highlighting this excellent, research paper. Findings from this paper have been integrated and included within the ‘Eligibility of population or types of participants’ section of the review.

**7. What types of study designs are considered for inclusion?**

 We have now clarified and will include the following types of study designs. All peer review empirical research, qualitative, quantitative, mixed methods, and action research studies will be included in this review. See Table 1. Inclusion and exclusion criteria.

**8. Are you able to provide examples of quality care metrics?**

 Thank you for this suggestion. We have in version 2 of the review. We included examples of quality care metrics from public health nursing which may be relevant to general practice nursing. We have also included examples of metrics developed for ambulatory care nurses in the USA. See the section titled ‘Nursing Quality Care Process Metrics’.

**9. Please clarify if advanced practice nurses/nurse practitioners or other classes of registered nursing are included/excluded within your review.**

 Research involving all classes of registered nurses will be included in this scoping review, including advanced nurse practitioners. This is now clarified in the ‘Eligibility of population or types of participants’ section.

**10. One of your objectives is to examine the use of QCM in providing advantages for patient outcomes and/or service delivery – however, your inclusion/exclusion criteria do not include anything related to reporting of outcomes within studies. Also, wouldn't this objective lend itself to a systematic review rather than a scoping review?**

 Thank you for highlighting the potential difficulties surrounding reporting outcomes in this scoping review. We have considered your comments and agree with your observations re scoping v systematic review. Therefore outcome reporting has been omitted from the objectives of this review.

**11. Have you considered using Covidence?**

**​​​​​​​**We will use Covidence, and this is referred to in the ‘Stage 3: Study selection’ section of the document.

**12. Please comment on how the quality of studies will be assessed.**

 It is our understanding that scoping reviews aim to map the extent of evidence and do not seek to report on the quality of evidence as referred to by Munn ^1^. However, the PAGER framework for improving the quality of scoping review reporting will be used to ensure a consistent clear approach to analysis and reporting of the review findings ^2^.

**References.** 

 1.  Munn, Z., et al., *Systematic review or scoping review? Guidance for authors when choosing between a systematic or scoping review approach.* BMC Medical Research Methodology, 2018. **18**(1): p. 143.

 2.   Bradbury-Jones, C., et al., *Scoping reviews: the PAGER framework for improving the quality of reporting.* International Journal of Social Research Methodology, 2021: p. 1-14.
